# Application of Traditional Vaccine Development Strategies to SARS-CoV-2

**Published:** 2022-08-16

**Authors:** Halie M. Rando, Ronan Lordan, Alexandra J. Lee, Amruta Naik, Nils Wellhausen, Elizabeth Sell, Likhitha Kolla, COVID-19 Review Consortium, Anthony Gitter, Casey S. Greene

**Affiliations:** Department of Systems Pharmacology and Translational Therapeutics, University of Pennsylvania, Philadelphia, Pennsylvania, United States of America; Department of Biochemistry and Molecular Genetics, University of Colorado School of Medicine, Aurora, Colorado, United States of America; Center for Health AI, University of Colorado School of Medicine, Aurora, Colorado, United States of America; Institute for Translational Medicine and Therapeutics, Perelman School of Medicine, University of Pennsylvania, Philadelphia, PA 19104-5158, USA; Department of Medicine, Perelman School of Medicine, University of Pennsylvania, Philadelphia, PA 19104, USA; Department of Systems Pharmacology and Translational Therapeutics, Perelman School of Medicine, University of Pennsylvania; Philadelphia, PA 19104, USA; Department of Systems Pharmacology and Translational Therapeutics, University of Pennsylvania, Philadelphia, Pennsylvania, United States of America; Children’s Hospital of Philadelphia, Philadelphia, PA, United States of America; Department of Systems Pharmacology and Translational Therapeutics, University of Pennsylvania, Philadelphia, Pennsylvania, United States of America; Perelman School of Medicine, University of Pennsylvania, Philadelphia, Pennsylvania, United States of America; Perelman School of Medicine, University of Pennsylvania, Philadelphia, Pennsylvania, United States of America; Department of Biostatistics and Medical Informatics, University of Wisconsin-Madison, Madison, Wisconsin, United States of America; Morgridge Institute for Research, Madison, Wisconsin, United States of America; Department of Systems Pharmacology and Translational Therapeutics, University of Pennsylvania, Philadelphia, Pennsylvania, United States of America; Childhood Cancer Data Lab, Alex’s Lemonade Stand Foundation, Philadelphia, Pennsylvania, United States of America; Department of Biochemistry and Molecular Genetics, University of Colorado School of Medicine, Aurora, Colorado, United States of America; Center for Health AI, University of Colorado School of Medicine, Aurora, Colorado, United States of America

## Abstract

Over the past 150 years, vaccines have revolutionized the relationship between people and disease. During the COVID-19 pandemic, technologies such as mRNA vaccines have received significant attention due to their novelty and successes. However, more traditional vaccine development platforms have also been applied against SARS-CoV-2, yielding important tools in the worldwide fight against the virus.

A variety of approaches have been used to develop COVID-19 vaccines that are now authorized for use in countries around the world. In this review, we highlight strategies that focus on the viral capsid outwards, rather than on the nucleic acids inside. Such approaches broadly fall into two categories: whole-virus vaccines and subunit vaccines. Whole-virus vaccine approaches use the virus itself, either in an inactivated or attenuated state. Subunit vaccines isolate an immunogenic component of the virus using various strategies that is then introduced through vaccination. We highlight specific vaccine candidates that utilize these approaches in different ways. In a companion manuscript, we review the more recent and novel development of nucleic-acid based vaccine technologies.

We further consider the role that these COVID-19 vaccine development programs have played in providing immunity to people around the world. Well-established vaccine technologies have proved especially important because of the significant role they have played in COVID-19 vaccine access at the global scale. Vaccine development programs that use established platforms have been undertaken in a much wider range of countries than those using nucleic-acid-based technologies, which have been led by wealthy Western countries. Therefore, these vaccine platforms, while less cutting-edge on the biotechnology side, have proven to be extremely important to the management of SARS-CoV-2.

## 2 Importance

As of August 14, 2022, there have been over 590,339,339 SARS-CoV-2 cases, and the virus has taken the lives of at least 6,435,645 people globally. The development, production, and distribution of vaccines is imperative to saving lives, preventing illness, and reducing the economic and social burdens caused by the COVID-19 pandemic. Much emphasis has been placed on vaccines that use cutting-edge biotechnology. However, more traditional methods of vaccine development that were refined throughout the twentieth century have been critical in increasing vaccine access worldwide. Effective deployment is necessary to reducing the susceptibility of the world’s population, which is especially important in light of emerging variants. In this review, we discuss the safety, efficacy, and distribution of vaccines developed using established technologies. In a separate review, we describe the vaccines developed using nucleic acid-based vaccine platforms. From the current literature, it is clear that the well-established vaccine technologies are also highly effective against SARS-CoV-2 and are being used to address the challenges of COVID-19 globally, including in low- and middle-income countries. This worldwide approach is critical for reducing the devastating impact of SARS-CoV-2.

## 3 Introduction

The development of vaccines is widely considered one of the most important medical advances in human history. Over the past 150 years, several approaches to vaccines have been developed and refined [1]. The COVID-19 pandemic has generated atypical circumstances compared to past health crises, leading to differences in vaccine development against this pathogen. One way in which the COVID-19 pandemic differs from prior global health crises is that the SARS-CoV-2 viral genome was sequenced, assembled, and released very early on (January 2020). This genomic information has been important in shaping the biomedical response to this pathogen [2,3]. All the same, vaccines have been developed since long before the concept of a virus or a viral genome was known, and as early as September 2020, there were over 180 vaccine candidates against SARS-CoV-2 in development, many of which used more traditional vaccine technologies [4]. However, media attention has largely focused on vaccine development platforms that have produced vaccine candidates using new technologies, especially mRNA vaccines due to their rapid deployment. We review vaccine technologies used for SARS-CoV-2 in two parts: here, the application of traditional vaccine development platforms to SARS-CoV-2, and separately, nucleic acid-based approaches [5].

Understanding vaccine development programs that are using well-established technologies is important for a global perspective on COVID-19. As of August 15, 2022, 41 SARS-CoV-2 vaccines have been approved for use in at least one country. A resource tracking the distribution of 29 vaccines indicates that, as of August 15, 2022, doses have been administered across 223 countries [6]. Many of these vaccines use platforms that do not require information about the viral genome, with 17 developed using subunit and 11 using whole-virus approaches. The types of vaccines available varies widely throughout the world, as the process of developing and deploying a vaccine is complex and often requires coordination between government, industry, academia, and philanthropic entities [7].

Another difference between prior global health crises and the COVID-19 pandemic is the way that vaccines are evaluated. A vaccine’s success is often discussed in terms of vaccine efficacy (VE), which describes the protection conferred during clinical trials [8]. The real-world protection offered by a vaccine is referred to as its effectiveness [8]. Additionally, protection can mean different things in different contexts. In general, the goal of a vaccine is to prevent disease, especially severe disease, rather than infection itself. As a proxy for VE, vaccine developers often test their candidates for serum neutralizing activity, which has been proposed as a biomarker for adaptive immunity in other respiratory illnesses [9]. The duration and intensity of the COVID-19 pandemic has made it possible to test multiple vaccines in phase III trials, where the effect of the vaccines on a cohort’s likelihood of contracting SARS-CoV-2 can be evaluated, whereas this has not always been feasible for other infectious diseases that have been controlled more quickly. Estimates of VE have been released for many vaccine candidates across a number of technology types based on phase III trial data.

However, efficacy is not a static value, and real-world efficacy can vary with location and over time. Shifts in efficacy have been an especially heightened topic of concern since late 2021 given the potential for variants of SARS-CoV-2 to influence VE. Due to viral evolution, vaccine developers are in an arms race with a pathogen that benefits from mutations that reduce its susceptibility to adaptive immunity. The evolution of several variants of concern (VOC) presents significant challenges for vaccines developed based on the index strain identified in Wuhan in late 2019. We discuss these variants in depth elsewhere [10]. To date, the most significant VOC identified are Alpha (2020), Beta (2020), Gamma (2020), Delta (2021), and Omicron (2021), with various subvariants of Omicron being the most recently identified (2022). The efficacy of vaccines in the context of these variants is discussed where information is available.

While the relationship between a vaccine and a pathogen is not static, the data clearly demonstrates that a variety of efficacious vaccines have been developed against SARS-CoV-2. Here we discuss a selection of programs that use well-established vaccine biotechnologies. These programs have been undertaken worldwide, in complement to the more cutting-edge approaches developed and distributed in the United States (U.S.), the European Union (E.U.), the United Kingdom (U.K.), and Russia [5]. In this review, we discuss vaccine development using two well-established technologies, whole-virus vaccination and subunit vaccination, and the role these technologies have played in the global response to the COVID-19 pandemic.

## 4 Whole-Virus Vaccines

Whole-virus vaccines have the longest history among vaccine development approaches. Variolation, which is widely considered the first vaccination strategy in human history, is one example [11,12]. Famously, variolation was employed against smallpox when healthy individuals were exposed to pus from an individual infected with what was believed to be either cowpox or horsepox [11,12,13,14]. This approach worked by inducing a mild case of a disease. Therefore, while whole-virus vaccines confer adaptive immunity, they also raise safety concerns [13,15,16]. As of 2005, most vaccines still used whole-virus platforms [17], and these technologies remain valuable tools in vaccine development today [1]. Whole virus vaccine candidates have been developed for COVID-19 using both live attenuated viruses and inactivated whole viruses.

### 4.1 Live-Attenuated Virus Vaccines

Live-attenuated virus vaccines (LAV), also known as replication-competent vaccines, use a weakened, living version of a disease-causing virus or a version of a virus that is modified to induce an immune response [4]. Whether variolation is the first example of a LAV being used to induce immunity is debated [1,15]. The first deliberate (albeit pathogen-naïve) attempt to develop an attenuated viral vaccine dates back to Louis Pasteur in 1885. The next intentional LAVs were developed against the yellow fever virus in 1935 and influenza in 1936 [18].

Early efforts in LAV development relied on either the identification of a related virus that was less virulent in humans (e.g., cowpox/horsepox or rotavirus vaccines) or the culturing of a virus *in vitro* [1,13]. Today, a virus can be attenuated by passaging it in a foreign host until, due to selection pressure, the virus loses its efficacy in the original host. Alternatively, selective gene deletion or codon deoptimization can be utilized to attenuate the virus [4], or foreign antigens can be integrated into an attenuated viral vector [19]. LAVs tend to be restricted to viral replication in the tissues around the location of inoculation [18], and some can be administered intranasally [4].

Today, LAVs are used globally to prevent diseases caused by viruses such as measles, rubella, polio, influenza, varicella zoster, and the yellow fever virus [20]. There were attempts to develop LAVs against both SARS-CoV-1 and MERS-CoV [21], but no vaccines were approved. It is generally recognized that LAVs induce an immune response similar to natural infection, and they are favored because they induce long-lasting and robust immunity that can protect from disease. This strong protective effect is induced in part by the immune response to the range of viral antigens available from LAV, which tend to be more immunogenic than those from non-replicating vaccines [15,21,22].

### 4.2 Application to COVID-19

To date, LAVs have not been widely deployed against SARS-CoV-2 and COVID-19. All the same, there are several COVID-19 LAV candidates in the early (preclinical/phase I) stages of investigation. These candidates utilize different approaches.

One candidate in the preclinical stage is YF-S0, a single-dose LAV developed at Belgium’s Katholieke Universiteit Leuven that uses live-attenuated yellow fever 17D (YF17D) as a vector for a noncleavable prefusion conformation of the SARS-CoV-2 antigen [19]. YF-S0 induced a robust immune response in three animal models and prevented SARS-CoV-2 infection in macaques and hamsters [19].

Other programs are developing codon deoptimized LAV candidates [23,24,25]. This approach follows the synthetic attenuated virus engineering (SAVE) strategy to select codon substitutions that are suboptimal for the virus [25,26]. New York-based Codagenix and the Serum Institute of India reported a successful preclinical investigation [25] of an intranasally administered deoptimized SARS-CoV-2 LAV known as COVI-VAC, and COVI-VAC entered phase I human trials and dosed its first participants in January 2021 [24,27]. This vaccine is optimized through the removal of the furin cleavage site (see [2] for a discussion of this site’s importance) and deoptimization of 283 codons [28]. The results of the COVI-VAC phase I human trials are expected soon.

Another company, Meissa Vaccines in Kansas, U.S., which also develops vaccines for respiratory syncytial virus (RSV), has developed an intranasal live-attenuated chimeric vaccine. Chimeric vaccines integrate genomic content from multiple viruses to create a more stable LAV [29]. Enrollment for phase I human trials began in March 2021 and recruitment is ongoing [24,30].

Finally, Bacillus Calmette-Guerin (BCG) vaccines that use LAVs are being investigated for the prophylaxis of COVID-19 (see online Appendix [31]). The purpose of the BCG vaccine is to prevent tuberculosis, but non-specific effects against other respiratory illnesses have suggested a possible benefit against COVID-19 [32]. However, a multicenter trial that randomly assigned participants 60 years and older to vaccination with BCG (n = 1,008) or placebo (n = 1,006) found that BCG vaccination had no effect on the incidence of SARS-CoV-2 or other respiratory infections over the course of 12 months [33]. Despite these negative findings, BCG vaccination did induce a stronger antibody and cytokine response following COVID-19 infection. Currently, investigations of BCG vaccines against COVID-19 are being sponsored by institutes in Australia in collaboration with the Bill and Melinda Gates Foundation [34] and by Texas A&M University in collaboration with numerous other U.S. institutions [35].

Safety data is not yet available for human studies of LAV COVID-19 vaccines. In general, though, safety concerns previously associated with LAV have been largely mitigated in the modern manufacturing process. Manufacturers use safe and reliable methods to produce large quantities of vaccines once they have undergone rigorous preclinical studies and clinical trials to evaluate their safety and efficacy. However, one remaining safety concern may be contributing to the relatively slow emergence of LAV candidates against COVID-19: they still may present risk to individuals who are immunocompromised [36], which is an even greater concern when dealing with a novel virus and disease. Additional data are needed to ascertain how this technology performs in the case of SARS-CoV-2. Despite the long and trusted history of LAV development, this vaccine strategy has not been favored against COVID-19, as other technologies have shown greater expediency and safety compared to the time-consuming nature of developing LAVs for a novel virus.

### 4.3 Response to Variants of Concern

While no phase III trial data is available for LAV vaccine candidates, some manufacturers have proactively sought to respond to the emergence of VOC. For example, a poster reported that Syrian golden hamsters who received COVI-VAC were significantly less likely to lose weight following viral challenge with the Beta VOC [28]. Additionally, the protective effect of YF-S0 against VOC has been investigated in hamsters [37]. Even for a small number of hamsters that developed breakthrough infections after exposure to the index strain or the Alpha variant, viral loads were very low [37]. However, much higher rates of breakthrough infection and higher viral loads were observed when the hamsters were exposed to the Beta variant [37]. Reduced seroconversion and neutralizing antibody (nAb) titers were also observed against the Beta and Gamma variants [37]. As a result, a modified version of YF-S0, called YF-S0*, was developed to include a modified spike protein intended to increase immunogenicity by including the full spectrum of amino acids found in the Gamma VOC as well as stabilizing the S protein’s conformation [37]. No breakthrough infections were observed following vaccination with YF-S0 and exposure to the index strain and the Alpha, Beta, Gamma, and Delta variants [37]. YF-S0* also reduced viral shedding for several VOC relative to YF-S0 [37], and it was also observed to produce significantly more nAbs against the Omicron variant [37]. Therefore, while data from human studies are not available, preclinical results suggest that LAV vaccines likely confer some protection against VOC even when designed with the index strain. Modifications to the design may make this protection more robust as SARS-CoV-2 evolves.

## 5 Inactivated Whole-Virus Vaccines

Inactivated whole-virus (IWV) vaccines are another well-established vaccine platform. This platform uses full virus particles generally produced via cell culture that have been rendered non-infectious by chemical (i.e., formaldehyde or β-propiolactone [38]) or physical (i.e., heat or ultraviolet radiation) means. In general, these vaccines mimic the key properties of the virus that stimulate a robust immune response, but the risk of adverse reactions is reduced because the virus is inactivated and thus unable to replicate. Though these viral particles are inactivated, they retain the capacity to prime the immune system. The size of the viral particle makes it ideal for uptake by antigen-presenting cells, which leads to the stimulation of helper T-cells [39]. Additionally, the array of epitopes on the surface of the virus increases antibody binding efficiency [39]. The native conformation of the surface proteins, which is also important for eliciting an immune response, is preserved using these techniques [40]. Membrane proteins, which support B-cell responses to surface proteins, are also induced by this method [41].

IWV vaccines have been a valuable tool in efforts to control many viruses. Some targets of IWV vaccines have included influenza viruses, poliovirus, and hepatitis A virus. Inactivated vaccines can generally be generated relatively quickly once the pathogenic virus has been isolated and can be passaged in cell culture [21,42]; the fact that these vaccines have not emerged as quickly as nucleic acid vaccines for SARS-CoV-2 may be due to significant investment in the infrastructure for nucleic acid vaccine technologies [43], which are more modular and immunogenic [5].

Past applications to human coronaviruses (HCoV) have focused predominantly on SARS-CoV-1. Preclinical studies have demonstrated that IWV SARS-CoV-1 vaccine candidates elicited immune responses *in vivo*. These vaccines generated nAb titers at concentrations similar to those evoked by recombinant protein vaccines [40,44]. Studies in ferrets and non-human primates demonstrated that IWV vaccines can offer protection against infection due to nAb and SARS-CoV-1-specific T cell responses [45]. However, several attempts to develop IWV vaccines against both SARS-CoV-1 and MERS-CoV were hindered by incidences of vaccine-associated disease enhancement (VADE) in preclinical studies [46]. In one example of a study in macaques, an inactivated SARS-CoV-1 vaccine induced even more severe lung damage than the virus due to an enhanced immune reaction [47]. Independent studies in mice also demonstrated evidence of lung immunopathology due to VADE in response to MERS-CoV IWV vaccination [48,49]. The exact mechanisms responsible for VADE remain elusive due to the specificity of the virus-host interactions involved, but VADE is the subject of investigation in preclinical SARS-CoV-2 vaccine studies to ensure the safety of any potential vaccines that may reach phase I trials and beyond [46].

### 5.1 Application to COVID-19

One of those, CoronaVac, was developed by Beijing-based biopharmaceutical company Sinovac. The developers inactivated a SARS-CoV-2 strain collected in China with β-propiolactone and propagated it using Vero cells [21]. The vaccine is coupled with an aluminum adjuvant to increase immunogenicity [21]. In phase I and II clinical trials, CoronaVac elicited a strong immunogenic response in animal models and the development of nAbs in human participants [53,54,55]. Administration followed a prime-boost regimen using a 0.5 ml dose containing 3 μg of inactivated SARS-CoV-2 virus per dose [56].

Results from a two-dose phase III trial following a 14-day prime boost became available in late 2020 [57], and an interim analysis identified specific IgG nAbs against the S1 receptor binding domain (RBD) and a robust IFN-γ secreting T cell response was induced via immunization with CoronaVac [58]. Safety analysis of the CoronaVac vaccine during the phase II trial revealed that most adverse reactions were either mild (grade 1) or moderate (grade 2) in severity. In adults aged 18 to 59 years receiving a variety of dosage schedules, site injection pain was consistently the most common symptom reported [55]. In older adults, the most common local and systemic reactions were pain at the injection site (9%) and fever (3%), respectively [53]. In phase III trials, minimal side effects were reported [57].

Estimates of CoronaVac’s VE have varied across trials. CoronaVac demonstrated an efficacy of a little over 50% in Brazil, which was contested by Turkish officials claiming an efficacy of 91.25%, but ultimately after multiple announcements, the efficacy debate was settled at over 50% [59,60]. Subsequently, an interim analysis of the phase III randomized placebo-controlled trials conducted in Turkey enrolling 10,214 participants (approximately 2:1 vaccine:placebo) indicated efficacy of 83.5%, with minimal side effects reported [57], and a prospective national cohort study in Chile reported an adjusted estimated effectiveness of 66% for the prevention of COVID-19 with an estimated 90% and 87% prevention of hospitalization and death, respectively [61].

CoronaVac was first approved in China and has now been distributed in 63 countries across Africa, Asia, Europe, North America, and South America, including Brazil, Cambodia, Chile, Colombia, Laos, Malaysia, Mexico, Turkey, Ukraine, and Uruguay [6,62]. As of August 2021, Sinovac had reportedly produced over a billion doses of CoronaVac [62].

Two additional inactivated vaccine candidates were developed following a similar approach by the state-owned China National Pharmaceutical Group Co., Ltd., more commonly known as Sinopharm CNBG. One, known as BBIBP-CorV or Covilo, was developed in Beijing using the HB02 variant of SARS-CoV-2. At Sinopharm CNBG’s Wuhan Institute, a second vaccine was developed using the WIV04 variant of SARS-CoV-2 [63]. The viruses were purified, propagated using Vero cells, isolated, and inactivated using β-propiolactone [63,64]. Both vaccines are adjuvanted with aluminum hydroxide [63,64].

Preclinical studies indicated that Covilo induced sufficient nAb titers in mice, and a prime-boost immunization scheme of 2 μg/dose was sufficient to protect rhesus macaques from disease [64]. For the other vaccine, nAbs were detected in all groups 14 days after the final dose in the phase I part of the trial [65], with similar findings reported in interim phase II data [65]. In phase II trials, the BBIBP-CorV vaccine appeared well-tolerated, with 23% of participants in the vaccine condition (482 total participants, 3:1, vaccine:placebo) reporting at least one adverse reaction characterized as mild to moderate [66]. In phase III trials, Sinopharm CNBG’s Covilo vaccine made from the WIV04 strain achieved an efficacy of 72.8% and was well tolerated [67]. Sinopharm affiliates in the UAE in early December 2020 claimed the vaccine had 86% efficacy, which was later at odds with a Sinopharm Beijing affiliate that stated that Covilo had a 79.34% efficacy later that same month [68].

Other vaccine development programs have been led through industry partnerships with governmental organizations. Bharat Biotech International Ltd., which is the biggest producer of vaccines globally, collaborated with the Indian Council of Medical Research (ICMR) National Institute of Virology (NIV) to develop another IWV, Covaxin, which is also known as BBV152. Preclinical studies of Covaxin in hamsters [69] and macaques [70] indicated that the vaccine induced protective responses deemed sufficient to move forward to human trials. Phase I and phase I/II studies indicated that Covaxin adjuvanted with alum and a Toll-like receptor 7/8 (TLR7/8) agonist was safe and immunogenic and that it induced Th1-skewed memory T-cell responses [71,72]. Only mild to moderate side-effects were reported upon immunization [71,72]. In July 2021, Covaxin’s overall vaccine efficacy was estimated at 77.8% for the prevention of COVID-19 based on a final enrollment of 25,798 people (approximately 1:1 vaccine:placebo) [73]. Only mild to moderate side-effects reported upon immunization [71,72], and in phase II trials, the BBIBP-CorV vaccine appeared well-tolerated, with 23% of participants in the vaccine condition (482 total participants, 3:1, vaccine:placebo) reporting at least one adverse reaction characterized as mild to moderate [66]. As of September 2021, Covaxin has been approved for emergency use in 30 countries across Africa, Asia, Europe, and South America, including Guyana, India, Iran, Zimbabwe, Nepal, Mauritius, Mexico, Nepal, Paraguay, and the Philippines [74].

Although IWV vaccine candidates were well-tolerated overall, both Sinovac’s CoronaVac and Sinopharm’s inactivated WIV04 vaccine trials were affected by concerns about adverse events. In CoronaVac’s trial of adults 18–59, 2% (n=7) of participants reported severe adverse events [53], causing the trial to be halted for investigation [75]. They were determined to be unrelated to the vaccine [53,75], which is now widely distributed. Similarly, a trial of the Sinopharm WIV04 vaccine in Peru was briefly paused due to safety concerns in relation to neurological symptoms [76], but this was later deemed unrelated to the vaccine, and the trial continued [77].

### 5.2 Real-World Safety and Efficacy

In the past, problems that arose during the manufacturing of IWV vaccines could present safety issues, but oversight of the manufacturing process has helped to improve IWV vaccine safety [78]. Nevertheless, the departure from norms necessitated by the COVID-19 crisis raised concerns about whether oversight would occur at pre-pandemic standards [78]. In general, the IWV COVID-19 vaccines have reported very few issues with safety. Additionally, safety audits have proactively identified concerns. For example, in April 2022, the WHO suspended procurement of Covaxin from Bharat Pharmaceuticals due to concerns about deviation from good manufacturing practice in their production facilities [79]. However, no safety issues had been reported in association with this vaccine. Rare cases of VADE have been reported in association with CoronaVac [80].

More concern has arisen around the issue of efficacy. One of the major limitations of IWV vaccines is their susceptibility to losing efficacy due to mutations in the epitopes of the circulating virus [16]. This loss of specificity over time is likely to be influenced by the evolution of the virus, and specifically by the rate of evolution in the region of the genome that codes for the antigen. The Beta variant appears to be more resistant to nAbs in sera from individuals immunized with Sinovac than the Alpha variant or wildtype virus, indicating that emerging variants may be of concern [81]. In agreement with previous studies demonstrating sera from individuals vaccinated with Covaxin efficiently neutralized the Alpha variant (B.1.1.7) and the Delta variant (B.1.617.2) [82,83,84], the phase III trial reported a 65.2% efficacy against the Delta variant (B.1.617.2) [73]. However, studies suggested the Beta variant was more resistant (compared to the wildtype and Alpha variants) to nAbs in sera from individuals immunized with Sinovac [81]. Another preprint determined that sera from individuals immunized with Covaxin had effective nAbs against the Delta variant and the so-called Delta plus variant (AY.1) [82]. Indeed, sera obtained from Covaxin boosted individuals (n = 13) [85] and those who were vaccinated with Covaxin but recovered from a breakthrough infection (n=31) also neutralized the Omicron variant [86].

Notably, a preprint reported that antisera (i.e., the antibody-containing component of the sera) from 12 people immunized with BBIBP-CorV exhibited nAb capacity against the Beta variant (B.1.351), wild type SARS-CoV-2 (NB02), and one of the original variants of SARS-CoV-2 (D614G) [87]. Another preprint including sera from 282 participants used a surrogate neutralizing assay, a test that generally correlates with nAbs, to determine that Covilo appears to induce nAbs against the binding of the RBD of wild type SARS-CoV-2 and the Alpha, Beta, and Delta variants to ACE2 [88]. Indeed, a study showed that the Alpha variant exhibited very little resistance to neutralization by sera of those immunized with Covilo, but the Beta variant was more resistant to neutralization by almost a factor of 3 [81]. The authors noted that no evidence of VADE was detected using this vaccine in phase II data [65].

Concern was raised about the efficacy of CoronaVac following reports that over 350 doctors became ill with COVID-19 in Indonesia despite being immunized with CoronaVac [89]. In addition to concerns raised by the evolution of SARS-CoV-2, it is important to consider the duration of immunity over time. Studies are underway to determine whether a booster immunization is required for several IWV vaccines, including CoronaVac [90] and Covaxin [91]. A phase I/II clinical trial of CoronaVac in an elderly cohort (adults 60 years and older) in China determined that by 6 to 8 months following the second dose, nAb titers were detected below the seropositive cutoff [92]. One preprint has reported that 6 months after the second vaccination, a booster dose of CoronaVac markedly increased geometric mean titers of SARS-CoV-2 nAbs [93]. However, the reduction of nAbs was ameliorated by a booster dose administered 8 months after the second CoronaVac dose. A preprint study of healthcare workers in China has since indicated that a booster shot of Covilo elevates B cell and T cell responses and increases nAb titers [94]. In May 2021, the UAE announced it would consider booster shots for all citizens who had been immunized with Covilo, which was shortly followed by a similar announcement in Bahrain, and by August 29, 2021, the UAE mandated booster shots for all residents who had received Covilo [62].

Additionally, heterologous vaccine boosters are also being considered in many cases. Indeed, Chinese [95] and Chilean [96] researchers have opted to investigate options to administer different vaccines (e.g., an mRNA vaccine dose) as a booster dose to individuals who have already received two doses of the IWV vaccine CoronaVac. Another study determined that using a viral-vectored vaccine (CanSino’s Convidecia) or an mRNA vaccine (Pfizer/BioNTech’s BNT162b2) instead of CoronaVac in a prime-boost vaccination regimen could induce a more robust immune response [97,98]. Today, booster immunization is suggested for several whole-virus vaccines.

## 6 Subunit Vaccines

Efforts to overcome the limitations of live-virus vaccines led to the development of approaches first to inactivate viruses (circa 1900), leading to IWV vaccines, and then to purifying proteins from viruses cultured in eggs (circa 1920) [1,99]. The purification of proteins then set the stage for the development of subunit vaccines based on the principle that the immune system can be stimulated by introducing one or more proteins or peptides isolated from the virus. Today, such approaches often use antigens isolated from the surface of the viral particle that are key targets of the immune system (protein subunit vaccines). Advances in biological engineering have also facilitated the development of approaches like viral-like particle (VLP) vaccines using nanotechnology [100]. VLPs share the conformation of a virus’s capsid, thereby acting as an antigen, but lack the replication machinery [101]. Both protein subunit and VLP vaccines thus mimic the principle of whole virus vaccines but lack the genetic material required for replication, removing the risk of infection [102].

Unlike whole-virus vaccines, which introduce the whole virus, subunit vaccines stimulate the immune system by introducing one or more proteins or peptides of the virus that have been isolated. The main advantage of this platform is that subunit vaccines are considered very safe, as the antigen alone cannot cause an infection [103]. Both protein subunit and VLP vaccines thus mimic the principle of whole virus vaccines but lack the genetic material required for replication, removing the risk of infection [102]. Protein subunit vaccines can stimulate antibodies and CD4^+^ T-cell responses [101,104].

The subunit approach is also favored for its consistency in production. The components can be designed for a highly targeted immune response to a specific pathogen using synthetic immunogenic particles, allowing the vaccine to be engineered to avoid allergen and reactogenic sequences [105]. One limitation is that, in the case of protein subunit vaccines, adjuvants are usually required to boost the immune response [106] (see online Appendix [31]). These adjuvants are immunogenic substances, which include, for example, alum (aluminum hydroxide), squalene- or saponin-based adjuvants, and Freund’s incomplete/complete adjuvants [105,107].

Protein subunit vaccine development efforts for both SARS-CoV-1 and MERS-CoV usually focused on the immunogenic RBD of the S protein [108,109,110]. The search for a potential SARS-CoV-1 vaccine included the development of vaccines targeting the full-length or trimeric S protein [111,112], those focused on the RBD protein only [108,109,110,113] or non-RBD S protein fragments [112,114], and those targeting the N and M proteins [115,116,117]. These efforts have been thoroughly reviewed elsewhere [118]. There have been examples of successful preclinical research including candidate RBD219N-1, a 218-amino-acid residue of the SARS-CoV-1 RBD that, when adjuvanted to aluminum hydroxide, was capable of eliciting a high RBD-specific and nAb response in both pseudovirus and live virus infections of immunized mice [119]. Other strategies investigating the potential use of the full length S DNA have also been investigated in mice and rhesus macaques, which elicited immune responses [120], but these responses were not as effective as the combination of S DNA and the S1 subunit protein together [120,121].

Similarly to the SARS-CoV-1 vaccine candidates, the MERS-CoV protein subunit vaccine candidates generally target the RBD [109,118,122,123,124,125], with some targeting the full length S protein [126], non-RBD protein fragments such as the SP3 peptide [127], and the recombinant N-terminal domain (rNTD) [128]. No protein subunit vaccine for MERS-CoV has progressed beyond preclinical research to date. VLPs have been investigated for development of vaccines against MERS and SARS [129,130] including testing in animal models [131,132], but once again, only preclinical data against HCoV has been collected [133]. However, protein subunit vaccines do play a role in public health and have contributed to vaccination against hepatitis B [134] and pertussis [135,136] since the 1980s and will likely continue to contribute to public health for the foreseeable future due to ongoing research in vaccines against influenza, SARS-CoV-2, Epstein-Barr virus, dengue virus, and human papillomavirus among others [137,138,139].

### 6.1 Application to COVID-19

**Figure 2:**
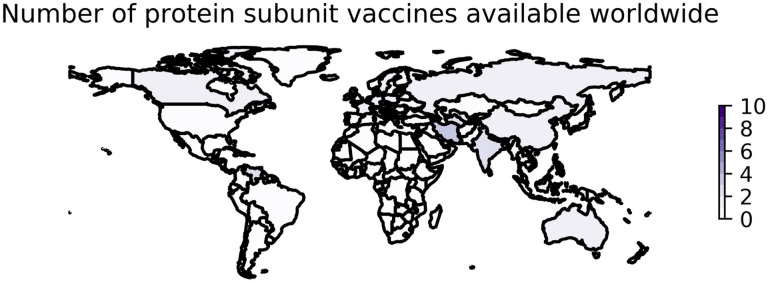
Worldwide availability of vaccines developed using protein subunit. This figure reflects the number of vaccines based on protein subunit technology that were available in each country as of August 15, 2022. These data are retrieved from Our World in Data [6,51] and plotted using geopandas [52]. See https://greenelab.github.io/covid19-review/ for the most recent version of this figure, which is updated daily.

The development of subunit vaccines against SARS-CoV-2 is a remarkable achievement given the short period of time since the emergence of SARS-CoV-2 in late 2019, particularly considering these types of vaccines have not played a major role in previous pandemics. More than 20 protein subunit vaccines from companies such as Sanofi/GlaxoSmithKline, Nanogen, and the Serum Institute of India have entered clinical trials for COVID-19 since the beginning of the pandemic [138], 17 have been approved, and at least 10 are being administered worldwide [6,140]. As of August 15, 2022, protein subunit vaccines are being distributed in at least 41 countries (Figure 2).

VLP vaccines have not progressed as rapidly. Programs seeking to develop VLP vaccines have used either the full-length S protein or the RBD of the S protein specifically as an antigen, although some use several different SARS-CoV-2 proteins [103]. As of August 15, 2022, only one VLP was available in one country (Canada) [6].

### 6.2 Nuvaxovid

One protein subunit vaccine for SARS-CoV-2 is NVX-CoV2373 or Nuvaxovid, produced by U.S. company Novavax and partners. NVX-CoV2373 is a protein nanoparticle vaccine constructed from a mutated prefusion SARS-CoV-2 spike protein in combination with a specialized saponin-based adjuvant to elicit an immune response against SARS-CoV-2. The spike protein is recombinantly expressed in Sf9 insect cells [141], which have previously been used for several other FDA-approved protein therapeutics [142], and contains mutations in the furin cleavage site (682-RRAR-685 to 682-QQAQ-685) along with two proline substitutions (K986P and V987P) that improve thermostability [141].

In preclinical mouse models, Novavax-CoV2373 elicited high anti-spike IgG titers 21 to 28 days post-vaccination that could neutralize the SARS-CoV-2 virus and protect the animals against viral challenge, with particularly strong effects when administered with the proprietary adjuvant Matrix-M^™^ [141]. In a phase I/II trial, a two-dose regimen of NVX-CoV2373 was found to induce anti-spike IgG levels and nAb titers exceeding those observed in convalescent plasma donated by symptomatic patients [143]. In line with the preclinical studies, the use of Matrix-M adjuvant further increased anti-spike immunoglobulin levels and induced a Th1 response. In a phase III randomized, observer-blinded, placebo-controlled clinical trial in 14,039 participants, two 5-μg doses of NVX-CoV2373 or placebo were administered 21 days apart in a 1:1 ratio from late September to late November 2020 [144].

In the phase III trial, the efficacy of Novavax’s Nuvaxovid was reported to be 89.7%, with a total of 10 patients developing COVID-19 in the vaccine group versus 96 in the placebo group [144]. No hospitalizations or deaths were reported in the vaccine group. An additional phase III randomized, observer-blinded, placebo-controlled trial was conducted in the U.S. and Mexico, enrolling 29,949 participants and administering at least 1 vaccine in a 2:1 ratio from late December 2020 to late February 2021 with the same primary endpoints as the U.K. trial [145]. A vaccine efficacy of 90.4% was reported based on 77 cases total, 63 of which occurred in the placebo group. All moderate to severe cases of COVID-19 occurred in the placebo group.

The conclusions of both trials indicate that the NVX-CoV2373 vaccine is safe and effective against COVID-19. In both trials, the vaccine was found to be well-tolerated [144,145]. The Novavax vaccine has since been authorized for use in several places, including the United Kingdom [146], the E.U. [147], and the U.S. [148].

### 6.3 Covifenz

The leading example of a VLP approach applied to COVID-19 comes from Covifenz, developed by Canadian company Medicago [149]. This vaccine was developed using plant-based VLP technology [150] that the company had been investigating in order to develop a high-throughput quadrivalent VLP platform to provide protection against influenza [151]. The approach utilizes *Nicotiana benthamiana*, an Australian relative of the tobacco plant, as an upstream bioreactor [151,152]. Specifically, the *S* gene from SARS-CoV-2 in its prefusion conformation is inserted into a bacterial vector (*Agrobacterium tumefaciens*) that then infects the plant cells [151,152]. Expression of the S glycoprotein causes the production of VLPs composed of S trimers anchored in a lipid envelope that accumulate between the plasma membrane and the cell wall of the plant cell [152]. Because these VLPs do not contain the SARS-CoV-2 genome, they offer similar advantages to whole-virus vaccines while mitigating the risks [151,152].

In the phase I study, 180 Canadian adults ages 18 to 55 years old were administered Covifenz as two doses, 21 days apart, with three different dosages evaluated [152]. This study reported that when the VLPs were administered with an adjuvant, the vaccine elicited a nAb that was significantly (approximately 10 times) higher than that in convalescent sera [152]. The phase III trial examined 24,141 adults assigned to the treatment and control conditions at a 1:1 ratio between March and September of 2021 [153].

Covifenz was reported to be 71% effective in preventing COVID-19 in the per-protocol analysis [153]. Efficacy was only slightly lower in the intention-to-treat group at 69%, with the VE for the prevention of moderate-to-severe disease in this group estimated at 78.8%. Over 24,000 participants were included in the safety analysis, which reported that 92.3% of vaccine recipients reported local adverse events compared to 45.5% of placebo recipients, with rates for systemic adverse events at 87.3% and 65.0%, respectively. The adverse effects reported were generally mild to moderate, with the most common adverse effects being injection site pain, headache, myalgia, fatigue, and general discomfort. Only three patients (two in the vaccine group) reported grade 4 events, all after the second dose. The vaccine was approved for use in adults ages 18 to 65 by Health Canada in February 2022 [154].

**Figure 3:**
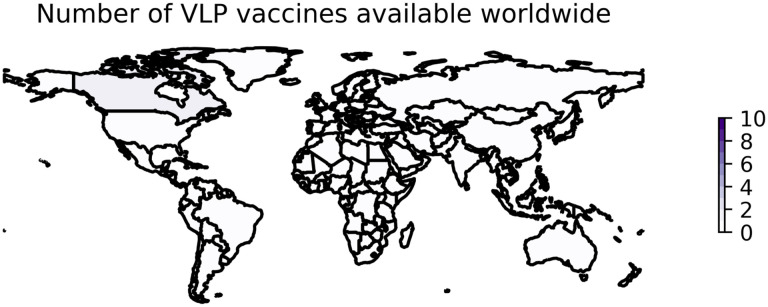
Worldwide availability of vaccines developed with VLPs. This figure reflects the number of vaccines based on VLP technology that were available in each country as of August 15, 2022. These data are retrieved from Our World in Data [51] and plotted using geopandas [52]. See https://greenelab.github.io/covid19-review/ for the most recent version of this figure, which is updated daily.

Plant-based expression systems such as this are relatively new [152] but are likely to offer unparalleled feasibility at scale given the speed and low-cost associated with the platform [155]. Additionally, the Covifenz vaccine can be stored at 2 to 8°C. However, the worldwide footprint of Covifenz, and of VLP-based technologies against SARS-CoV-2 broadly, remains small, with only one VLP vaccine approved for distribution in one country (Figure 3). Approval and administration of Covifenz in countries outside of Canada has been limited by concerns at the WHO about ties between Medicago and the tobacco industry [149,156]. While other species of plants have been explored as the upstream bioreactors for plant-derived VLPs, the specific species of tobacco used increased yield dramatically [157].

### 6.4 Real-World Safety and Efficacy

To date, data about the effect of viral evolution on the efficacy of subunit vaccines has been limited. *Post hoc* analysis in the phase III trial determined that the NovaVax vaccine had an efficacy of 86.3% against the Alpha variant (identified based on the presence/absence of the 69–70del polymorphism) and 96.4% against other variants [144]. In the second phase III trial [145], whole-genome sequencing was obtained from 61 of the 77 cases, and 79% of infections were identified as a VOC or variant of interest (VOI) that had been characterized at the time of the study. Vaccine efficacy against cases caused by VOC, among which the Alpha variant was predominant (88.6%), was reported to be 92.6% [145]. In late 2020, an analysis of efficacy in South African adults revealed an overall efficacy of 60.1% and a slightly lower efficacy of 50.1% against B.1.351 in particular [158]. Additionally, an analysis of a booster dose of NVX-CoV2373 administered six months after the primary series revealed a significant increase in neutralizing activity of VOC including Delta and Omicron [159]. It has also been reported that Novavax initiated booster trials in the U.K. [62].

Because the Covifenz results became available relatively recently (May 2022), limited data is available since the publication of phase III trial results [153]. However, it should be noted that the Covifenz trials were conducted in 2021, at a time during which the B.1.617.2 (Delta) and P.1 (Gamma) variants were predominant [153]. Genomic analysis of 122 out of 176 cases (165 in the per-protocol population) revealed that none of the COVID-19 cases reported were caused by the original Wuhan strain. Instead, 45.9% of cases were identified as the Delta variant, 43.4% as Gamma, 4.9% as Alpha, and 5.8% as VOIs. Therefore, the efficacy data from this phase III trial may be lower than it would have been if the trial had occurred earlier in the course of SARS-CoV-2’s evolution given that the S glycoprotein expressed in the VLPs was isolated from a 2020 sample of SARS-CoV-2 [153].

## 7 Global Vaccine Status and Distribution

The unprecedented deployment of COVID-19 vaccines in under a year from the identification of SARS-CoV-2 led to a new challenge: the formation of rapid global vaccine production and distribution plans. The development of vaccines is costly and complicated, but vaccine distribution can be just as challenging. Logistical considerations such as transport, storage, equipment (e.g., syringes), the workforce to administer the vaccines, and a continual supply from the manufacturers to meet global demands all must be accounted for and vary globally due to economic, geographic, and sociopolitical reasons [160,161,162]. As of August 9, 2022, at least 12.0 billion vaccine doses had been administered in at least 223 countries worldwide using 29 different vaccines [51]. The daily global vaccination rate at this time was 745.0 per million.

However, the distribution of these doses is not uniform around the globe. As of May 2022, vaccination rates remained lower in South America (3.1 per 100), Asia (1.9 per 100), Africa (0.3 per 100), and Oceania (0.1 per 100) than in North America, Europe, and Australia [163]. Vaccine production and distribution varies from region to region and seems to depend on the availability of the vaccines and potentially a country’s resources and wealth [164].

One effort to reduce these disparities is the COVID-19 Vaccines Global Access (COVAX) Facility, a multilateral initiative as part of the Access to COVID-19 Tools (ACT) Accelerator coordinated by the WHO, Gavi The Vaccine Alliance, and the Coalition for Epidemic Preparedness Innovations (CEPI), the latter two of which are supported by the Bill and Melinda Gates Foundation. Their intention is to accelerate the development of COVID-19 vaccines, diagnostics, and therapeutics and to ensure the equitable distribution of vaccines to low- and middle-income countries [165,166]. COVAX invested in several vaccine programs to ensure they would have access to successful vaccine candidates [167]. However, the initiative has been less successful than was initially hoped due to less participation from high-income countries than was required for COVAX to meet its goals [168].

**Figure 4:**
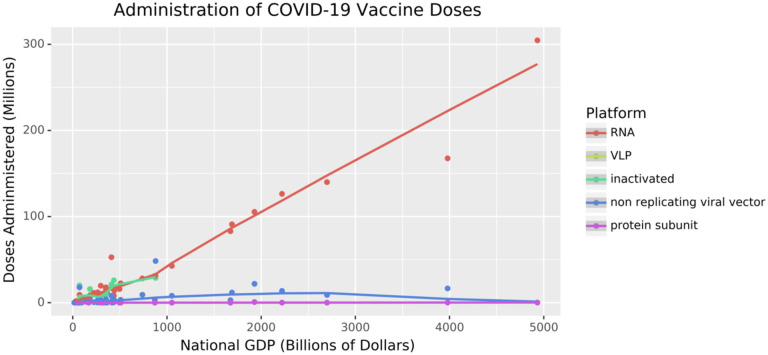
Vaccine Distribution across Platform Types as a Function of GDP. As of August 15, 2022, the number of doses distributed, by platform type, is shown as a function of GDP. These data are retrieved from Our World in Data [6,51] and plotted using the Python package plotnine [169]. Lines show a general trend in the data and are drawn using geom_smooth [170]. See https://greenelab.github.io/covid19-review/ for the most recent version of this figure, which is updated daily. Axes are not scaled per capita because both variables would be expected to be modulated by population size.

Additionally, the vaccine technologies available differ widely around the globe. As we review elsewhere [5], wealthier nations have invested heavily in mRNA and DNA vaccines. In contrast, as we describe above, many countries outside of Europe and North America have developed highly effective vaccines using more traditional approaches. As a result, there is a clear relationship between a country’s gross domestic product (GDP) and its access to vaccines (Figure 4).

When vaccines first became available, the wealthy nations of North America and Europe secured most of the limited COVID-19 vaccine stocks [171]. Throughout 2021, low- and middle-income countries faced steep competition with high-income countries for vaccines, and the rates of vaccination reflected this unequal distribution [172]. While the wealthiest countries in these regions could compete with each other for vaccines independent of programs such as COVAX [172], other countries in these regions have faced challenges in acquiring vaccines developed by the world’s wealthiest nations. Fortunately, while mRNA and DNA vaccine development programs are not widespread, vaccine development using whole-virus and subunit technologies has been undertaken worldwide. China and India, in particular, have developed several vaccines that are now widely available in these densely populated countries (see online Appendix [31]).

Still, many nations, especially in Africa, are reliant on the COVAX Facility, who have promised 600 million doses to the continent [173]. The COVAX plan seeks to ensure that all participating countries would be allocated vaccines in proportion to their population sizes. Once each country has received vaccine doses to account for 20% of their population, the country’s risk profile will determine its place in subsequent phases of vaccine distribution. However, several limitations of this framework exist, including that the COVAX scheme seems to go against the WHO’s own ethical principles of human well-being, equal respect, and global equity and that other frameworks might have been more suitable [174]. Furthermore, COVAX is supposed to allow poorer countries access to affordable vaccines, but the vaccines are driven by publicly traded companies that are required to make a profit [164].

In any case, COVAX provides access to COVID-19 vaccines that may otherwise have been difficult for some countries to obtain. COVAX aimed to distribute 2 billion vaccine doses globally by the end of 2021 [175]. According to Gavi, as of January 2022, COVAX had distributed over 1 billion vaccines to 144 participants of the program [176], short of its target but still a major global achievement. It is envisaged that COVAX may also receive additional donations of doses from Western nations who purchased surplus vaccines in the race to vaccinate their populations, which will be a welcome boost to the vaccination programs of low- and middle-income countries [177].

In general, deciding on the prioritization and allocation of the COVID-19 vaccines is also a challenging task due to ethical and operational considerations. Various frameworks, models, and methods have been proposed to tackle these issues with many countries, regions, or U.S. states devising their own distribution and administration plans [178,179,180,181,182]. The majority of the distribution plans prioritized offering vaccines to key workers such as health care workers and those who are clinically vulnerable, such as the elderly, the immunocompromised, and individuals with comorbidities, before targeting the rest of the population, who are less likely to experience severe outcomes from COVID-19 [183]. The availability of vaccines developed in a variety of countries using a variety of platforms is likely to work in favor of worldwide vaccine access.

## 8 Conclusions

Much attention has focused on the most novel vaccine technologies that have been deployed against SARS-CoV-2, but the established vaccine platforms discussed here have all made a significant impact on human health during the twentieth century and in some cases even earlier. The COVID-19 pandemic has demonstrated new potential in these established technologies. In the early 2000s, these technologies were explored for managing SARS-CoV-1 [184,185], but the epidemic was controlled before those efforts came to fruition [186]. Similarly, these technologies were explored for MERS-CoV, but outbreaks were sporadic and difficult to predict, making vaccine testing and the development of a vaccination strategy difficult [187]. However, in the COVID-19 pandemic, most of these technologies have been used to accelerate vaccine development programs worldwide. Therefore, they are also offering the opportunity to respond quickly to an emergent pathogen.

While these tried-and-true technologies don’t always produce vaccines with the highly desirable VE reported in mRNA clinical trials (which exceeded 90%), the efficacies are still very high, and these vaccines are extremely effective at preventing severe illness and death. As a result, the greater accessibility and stability of these vaccines makes them extremely valuable for the global effort to mitigate the loss of life from SARS-CoV-2.

## Figures and Tables

**Figure 1:**
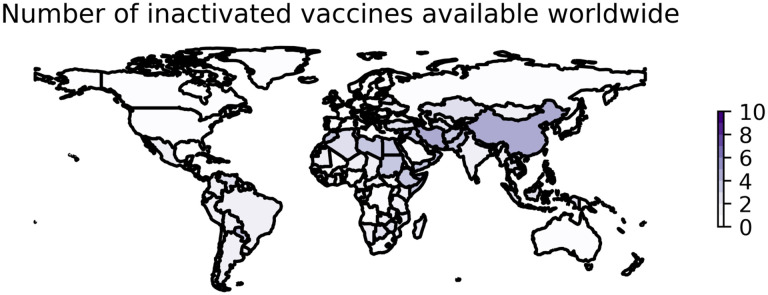
Worldwide availability of vaccines developed using inactivated whole viruses. This figure reflects the number of vaccines based on whole inactivated virus technology that were available in each country as of August 15, 2022. These data are retrieved from Our World in Data [51] and plotted using geopandas [52]. See https://greenelab.github.io/covid19-review/ for the most recent version of this figure, which is updated daily.

**Table 1:** Inactivated whole-virus vaccines approved in at least one country [50] as of August 15, 2022.

Vaccine	Company
Covaxin	Bharat Biotech
KoviVac	Chumakov Center
Turkovac	Health Institutes of Turkey
FAKHRAVAC (MIVAC)	Organization of Defensive Innovation and Research
QazVac	Research Institute for Biological Safety Problems (RIBSP)
KCONVAC	Shenzhen Kangtai Biological Products Co
COVIran Barekat	Shifa Pharmed Industrial Co
Covilo	Sinopharm (Beijing)
Inactivated (Vero Cells)	Sinopharm (Wuhan)
CoronaVac	Sinovac
VLA2001	Valneva

Several whole-virus vaccines have been developed against COVID-19 and are available in countries around the world (Table 1). As of August 15, 2022, 10 vaccines developed with IWV technology are being distributed in 116 countries (Figure 1).

**Table 2:** Subunit vaccines approved for use in at least one country [50] as of August 15, 2022.

Vaccine	Company	Platform
Zifivax	Anhui Zhifei Longcom	protein subunit
Noora vaccine	Bagheiat-allah University of Medical Sciences	protein subunit
Corbevax	Biological E Limited	protein subunit
Abdala	Center for Genetic Engineering and Biotechnology (CIGB)	protein subunit
Soberana 02	Instituto Finlay de Vacunas Cuba	protein subunit
Soberana Plus	Instituto Finlay de Vacunas Cuba	protein subunit
Covifenz	Medicago	VLP
MVC-COV1901	Medigen	protein subunit
Recombinant SARS-CoV-2 Vaccine (CHO Cell)	National Vaccine and Serum Institute	protein subunit
Nuvaxovid	Novavax	protein subunit
Razi Cov Pars	Razi Vaccine and Serum Research Institute	protein subunit
COVOVAX (Novavax formulation)	Serum Institute of India	protein subunit
SKYCovione	SK Bioscience Co Ltd	protein subunit
TAK-019 (Novavax formulation)	Takeda	protein subunit
SpikoGen	Vaxine/CinnaGen Co.	protein subunit
Aurora-CoV	Vector State Research Center of Virology and Biotechnology	protein subunit
EpiVacCorona	Vector State Research Center of Virology and Biotechnology	protein subunit
